# Participant factors associated with psychosocial impacts of lung cancer screening: A systematic review

**DOI:** 10.1002/cam4.70054

**Published:** 2024-08-03

**Authors:** Kathleen McFadden, Brooke Nickel, Nicole M. Rankin, Tong Li, Chloe J. Jennett, Ashleigh Sharman, Samantha L. Quaife, Nehmat Houssami, Rachael H. Dodd

**Affiliations:** ^1^ The Daffodil Centre The University of Sydney, a joint venture with Cancer Council NSW Sydney Australia; ^2^ School of Public Health, Faculty of Medicine and Health The University of Sydney Sydney Australia; ^3^ Melbourne School of Population and Global Health, Faculty of Medicine, Dentistry and Health Sciences The University of Melbourne Melbourne Australia; ^4^ Wolfson Institute of Population Health, Barts and The London School of Medicine and Dentistry Queen Mary University of London London UK

## Abstract

**Background:**

Psychosocial impacts of lung cancer screening (LCS) can cause both harm to individuals and serve as barriers to screening participation and adherence. Early data suggest that the psychosocial impacts of LCS are moderated by certain factors (e.g. sociodemographic characteristics and beliefs), but evidence synthesis is lacking. This systematic review aimed to understand individual‐level risk factors for psychosocial burden during LCS as a precursor to developing strategies to identify and support participants, and improve LCS engagement.

**Methods:**

Four databases were searched for full‐text articles published in English reporting any association between participant factors and psychosocial outcomes experienced during LCS. Study quality was assessed by two independent investigators; findings were synthesised narratively. The review was pre‐registered with PROSPERO and adhered to PRISMA guidelines.

**Results:**

Thirty‐five articles were included; most (33/35) studies were assessed at high or moderate risk of bias. Study designs were pre‐post (*n* = 13), cross‐sectional (*n* = 13), qualitative (*n* = 8) and mixed‐methods (*n* = 1) and conducted primarily in the United States (*n* = 17). Psychological burden in LCS varied, and was often associated with younger age, female gender, current smoking status or increased smoking history, lower education, lower socio‐economic group, not being married or co‐habiting and experience with cancer. However, results were mixed, and non‐significant associations were also reported across all factors. Beliefs (e.g. fatalism, stigma and expectation of LDCT results) and comorbid psychological burden were also linked to psychosocial outcomes, but evidence was sparse. Associations between risk perception, other participant factors and other psychosocial outcomes was inconclusive, likely reflecting individual biases in risk conceptualisation.

**Conclusion(s):**

Several participant factors are consistently reported to be associated with psychosocial impacts of LCS, though study heterogeneity and high risk of bias necessitate more robust evaluation. Further research on how perceptions, beliefs and expectations can be used to improve psychosocial outcomes during LCS is needed.

## 1 INTRODUCTION

Globally, lung cancer is the leading cause of cancer mortality.^1^ This is generally attributed to advanced‐stage diagnoses, making earlier detection of lung cancer a worldwide priority for cancer control. Low‐dose computed tomography (LDCT) screening for lung cancer, evaluated in landmark trials in the United States and Europe, can achieve a 20%–24% reduction in lung cancer mortality in high‐risk populations.^2^, ^3^ There is, however, concern that lung cancer screening (LCS) can induce psychological harm. Some psychological burden for participants is seen across most cancer screening programmes, primarily presenting as anxiety or distress to the individual or their family/carers, with consequences for screening participation, adherence and other medical help‐seeking behaviours as well.^4^, ^5^ For LCS, there are additional unique considerations for psychosocial outcomes. These include the potential for shame, regret or stigma around smoking behaviour, high chance of finding indeterminate nodules requiring surveillance and potential for overdiagnosis.^6^, ^7^

Current consensus is that LCS is unlikely to result in any clinically significant psychological burden for participants, and if it does, adverse effects will not persist long term.^4^, ^8^ However, evidence from real‐world LCS programmes is sparse, and most trial samples significantly under‐represent priority groups.^4^, ^8^ For example, participants in the Danish Lung Cancer Screening Trial were identified as likely being more ‘psychologically robust’ than a LCS population in a real‐world setting.^9^ Measuring psychosocial outcomes in communities where LCS would take place is particularly important for socially driven factors and outcomes (e.g. risk perception, stigma and peer pressure as motivation to quit smoking),^10^ given the difference in social processes and engagement between controlled trials and real‐world practice. Additionally, enrolment in an organised LCS programme would entail ongoing surveillance and/or regular screening, and so there may be changing or compounding psychosocial impacts over time. Measurement across these routine and follow‐up scans is limited as yet, and some study authors have acknowledged that long‐term psychosocial impacts may have been overlooked.^5^, ^9^

In addition to these considerations, evidence suggests that certain participant‐level risk factors significantly mediate or moderate psychosocial burden during LCS.^4^, ^5^, ^11^ These include sociodemographic characteristics, smoking status and history, and health beliefs, but a robust synthesis of the literature is needed. The aim of this systematic review is therefore to synthesise the evidence for individual factors associated with psychosocial outcomes of LCS. This would inform development of strategies for identifying and supporting participants who experience psychosocial harm through the LCS pathway. Additionally, psychological barriers to uptake have been connected to low rates of LCS,^12^, ^13^, ^14^ so a better understanding of related risk factors could support improved participation and adherence.

## 2 METHODS

### 2.1 Search strategy

Databases searched were Medline, Embase, PsycINFO and Cumulative Index of Nursing and Allied Health Literature (CINAHL) from inception to 15 June 2022. An update of the search was performed on 12 July 2023. In consultation with a research librarian, a search strategy for Medline was developed then modified to suit the required syntax for other databases (Table S1). No search limitations for date, language or geography were used. Searches of references and cited articles for all included studies were also conducted.

### 2.2 Study inclusion and exclusion criteria

Studies were included if they were original, full‐text articles reporting quantitative or qualitative psychosocial outcomes. Studies were excluded if they were reviews, case studies, case reports, opinions, comments or editorials. Participants in LCS via LDCT across the entire screening continuum (including enrolment, recruitment and follow‐up) were included; though studies where participants were only eligible for LCS (and had not engaged in the LCS pathway) were not.

Included factors and outcomes are presented in Box 1. Any psychosocial outcome was considered, including those related to decision‐making and smoking cessation (e.g. motivation and readiness to quit), though behavioural smoking outcomes (e.g. quit rates) were excluded. Only experienced outcomes were included, thus studies which reported on anticipated impacts of LCS were excluded. Relevant factors were any participant‐level predictor, moderator, mediator or covariate of an outcome of interest. Broadly, these were categorised into sociodemographic factors (e.g. age, gender and education level), health‐related factors (e.g. smoking history, experience with cancer and pre‐existing psychological burden), beliefs (e.g. risk perception, fatalism and stigma) and other factors. Stigma was included as a belief outcome, though noting that it has social aspects as well. There was cross‐over between factors and outcomes, with some categorised as both (e.g. certain beliefs).

BOX 1 **List of factors and outcomes examined in identified studies**

**Table:** 

Factors	Outcomes
Sociodemographic factors Gender Age Education Race/ethnicity Income, employment, insurance and deprivation Relationship status Health‐related factors Smoking status or history Pre‐ or co‐existing psychological burden Experience with cancer, lung cancer or lung cancer screening Health status Calculated lung cancer risk Beliefs Lung cancer risk perception (affective, comparative, absolute) Expectation of LDCT result Perception of health Fatalism Stigma Perception of LCS Other Social factors Informed decision‐making and knowledge Responses to COVID‐19	Psychological Worry, distress, stress and fear Anxiety Depression Health‐related quality of life (HRQoL) (Lung) cancer worry, distress, stress and fear LCS or LDCT‐specific worry, including concern/anxiety while waiting for LDCT results Guilt and shame Reassurance and relief Beliefs Lung cancer risk perception (affective, comparative, absolute) Perception of control Fatalism and perception of consequences Stigma Perception of LCS Decision‐related Decisional regret Decisional satisfaction Decisional conflict Smoking‐related Motivation/interest in quitting smoking Confidence/self‐efficacy to quit smoking Readiness to quit smoking Smoking worry Social Seeking social support

All relevant analyses within each study were reported; some studies examined multiple outcomes under a single category (e.g. anxiety and HRQoL under psychological outcomes), therefore results from a single study were sometimes conflicting within category summaries. Where a factor of interest was examined as a modifier or covariate, studies were included if the relationship between the factor of interest and the outcome was directly reported.

### 2.3 Data extraction and synthesis

Procedures for data synthesis and analysis were determined a priori. The lead investigator (KM) undertook title, abstract and full‐text screening, with a second investigator (BN or AS) independently assessing a 20% subset at both stages of screening to ensure agreement and consistency. Any discrepancies were discussed with reference to the pre‐defined inclusion and exclusion criteria, with consultation by a third investigator if required. Data extraction of study characteristics was performed by the lead investigator (KM) and checked for accuracy by another investigator (TL or CJJ). Results were extracted independently by two investigators (KM and either TL or CJJ). Evidence was summarised by factor and outcome of interest; given the heterogeneity across studies, meta‐analysis and subgroup analysis were not considered appropriate and instead results were synthesised narratively.

### 2.4 Quality assessment

Quality assessment was performed by two investigators independently (KM and either TL or CJJ), with any discrepancies resolved via discussion or via an additional investigator (BN). Validated tools from the Joanna Briggs Institute (JBI) were used and an overall assessment (low, moderate or high risk of bias) was given based on pre‐determined criteria about the number of appraisal checklist items fulfilled.^15^ Studies were not excluded from the review based on methodological quality, but this was considered in the interpretation of findings.

### 2.5 Protocol and registration

This systematic review presents the participant factors associated with psychosocial outcomes of LCS as part of a larger review (PROSPERO registration: CRD42022334634). A companion systematic review will report on the programme factors (service delivery aspects and interventions).

## 3 RESULTS

### 3.1 Study characteristics

**FIGURE 1:**
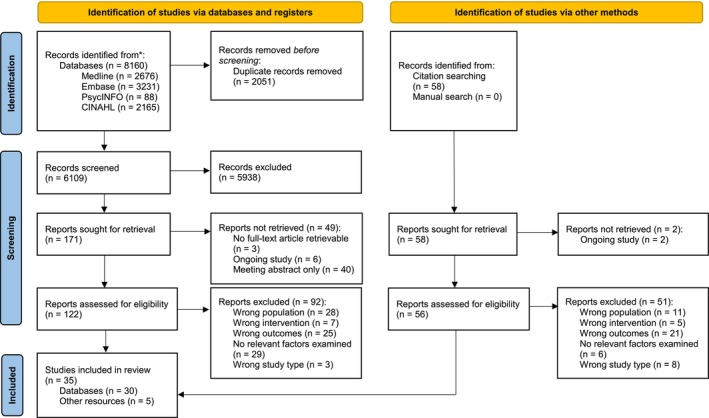
Results of search—PRISMA flow diagram.

Figure 1 presents the PRISMA flow diagram of search results.^16^ Key characteristics of the 35 included studies are presented in Table 1. Study designs included pre‐post studies (*n* = 13), cross‐sectional studies (*n* = 13), qualitative studies (*n* = 8) and mixed‐methods studies (*n* = 1), conducted in Australia (*n* = 1), Canada (*n* = 2), Denmark (*n* = 1), Netherlands/Belgium (*n* = 5), South Korea (*n* = 1), United Kingdom (*n* = 8) and United States (*n* = 17). Articles were published between 2001 and 2022.

**TABLE 1:** Key characteristics of included studies.

Author, year	Location (trial/programme)	Study design	Study aim	Participant description, *N*	Measurement point(s)	Factors tested	Outcomes of interest	Measurement tool	Risk of bias
Balata et al., 2020^43^	England (Manchester LHC pilot)	Pre‐post (cross‐sectional for outcomes of interest)	To determine if attendance at a Lung Health Check (LHC) and participation in LDCT influenced smoking behaviour and attitudes to smoking in a deprived population	Participants who enrolled in LHC pilot and attended a second LDCT *N* = 919 Currently smoking at T0 and T1: *N* = 415 Currently smoking at T0 and no longer smoking at T1: *N* = 47 No longer smoking at T0 and T1: *N* = 433 No longer smoking at T0 and currently smoking at T1: *N* = 24	T0 = Baseline (time of LHC) T1 = 1‐year post‐LHC (second LDCT)	Smoking status	Smoking worry (measured at T1)	3 items	Moderate
							Intention to quit smoking (measured at T1)	1 item	
							Confidence/self‐efficacy in quitting smoking (measured at T1)	2 items	
Barta et al., 2021^38^	United States	Cross‐sectional	To identify racial differences in attitudes and beliefs towards lung cancer and LCS, and determine whether sociodemographic factors/screening beliefs are associated with adherence rates to screening follow‐up	New LCS participants arriving for an SDM office visit prior to LDCT Study cohort: *N* = 269 HINTS cohort: *N* = 2235 (used only for cancer beliefs outcomes)	After recruitment, prior to SDM and LDCT	Race	Cancer beliefs (fatalism)	4 items taken from HINTS	High
							Risk perception	1 item	
							Lung cancer worry	3 items adapted from CWS	
							LDCT worry	3 items	
Bold et al., 2022^34^	United States	Cross‐sectional	To investigate patient characteristics, treatment perceptions and potential barriers to quitting smoking	Attendees for a lung cancer screening visit who reported current cigarette smoking *N* = 147	At time of LCS visit	Age Gender Race Nicotine dependence	Motivation/intention to quit smoking	1 item	High
							Concerns about quitting smoking	6 items	
Brain et al., 2016^20^	England (UKLS)	Pre‐post	To measure the effects of UKLS trial participation on short‐term and long‐term psychosocial outcomes	High‐risk individuals (≥5% over 5 years using the LLP_v2_ risk prediction model) randomly allocated to LDCT or control group At T0: *N* = 4055 Screening arm: *N* = 2018 Control arm: *N* = 2019	T0 = Baseline T1 = 2 weeks after LDCT result (or after assignment to control group) T2 = Mean time of 16 months after attending recruitment centre (range: 10–29 months)	Age Gender Ethnicity Education Socio‐economic group Marital status Smoking status Experience of lung cancer T0 cancer distress	Cancer distress (measured at T1, T2)	CWS	Moderate
Bunge et al., 2008^29^	Netherlands/Belgium (NELSON)	Pre‐post	To evaluate how many participants in a LCS trial had a low or high affective risk perception of developing lung cancer, and whether participants with a high affective risk perception showed a higher level of lung cancer‐specific distress during LCS	Participants who had a baseline LDCT appointment as part of the NELSON trial and received a negative or indeterminate scan result (positive results excluded from analysis) *N* = 351 T1 respondents: *N* = 324 T2 respondents: *N* = 288	T1 = 1 day before baseline screening T2 = 6 months after baseline screening (all results including from second screening round had been received)	Age Gender Smoking status Pack years Affective risk perception (feeling about risk)	HRQoL	SF‐12	High
							Affective risk perception (feeling about risk)	1 item	
							Lung cancer‐specific distress	IES	
Buttery et al., 2022^45^	England (Lung Health Check)	RCT (qualitative for outcome of interest)	To compare the effectiveness of two approaches to smoking cessation support for people who smoke attending a lung health screening service	Participants attending a targeted LHC randomised into either immediate smoking cessation support (with pharmacotherapy), or usual care (very brief advice to quit and signposting to local services) *N* = 84 (at follow‐up) Intervention: *N* = 48 Usual care: *N* = 36	3 months post intervention	Stress and anxiety Expectation of result Fear COVID‐19	Motivation to quit	Interviews	Moderate
Byrne et al., 2008^19^	United States (PLuSS)	Pre‐post	To measure the effect of screening outcomes on anxiety levels, fear of cancer and perceived risk of lung cancer for participants in three different screening outcome categories	Individuals recruited into the PLuSS trial T0 = 400 T3 = 341	T0 = Enrolment T1 = 1–2 weeks after receipt of CT result T2 = 6 months follow‐up T3 = 12 months follow‐up	Age Gender Race Marital status Education level Smoking status	Perceived risk of lung cancer	1 item	Moderate
							Anxiety (state, trait)	STAI	
							Fear of lung cancer	3 items adapted from PCQ	
Byrne et al., 2019^27^	United States	Cross‐sectional	To describe the characteristics of people being screened in community settings, including factors influencing screening decisions and the level of information sought prior to screening	Individuals undergoing LDCT‐based LCS in community‐based radiology clinics *N* = 27	After at least 1 LDCT	Age Gender Smoking status	Fear of being diagnosed with lung cancer	1 item per outcome (measured as importance to LCS decision‐making)	High
							Reassurance seeking		
							Perceived efficacy of LCS		
							Decisional conflict	DCS	
							Decisional satisfaction	Satisfaction with Decision Scale	
							Decisional regret	Decision Regret Scale	
Cordon et al., 2021^49^	United States (LSTH)	Mixed‐methods (qualitative and quantitative interview)	To explore the effects of COVID‐19 on older people who smoke enrolled in the Lung Screening, Tobacco and Health (LSTH) trial	Participants who had completed LDCT and were enrolled or had started a smoking cessation intervention (8 counselling sessions + 8 weeks of nicotine patches) or usual care (3 sessions + 2 weeks of patches) *N* = 30 Intervention: *N* = 21 UC: N = 9	During intervention or usual care session (some sessions were conducted before, and some during the COVID‐19 pandemic)	COVID‐19	Motivation to quit	Qualitative (interviews) Quantitative (1 item)	High (High for quantitative; Moderate for qualitative)
Dunn et al., 2017^23^	England (UKLS)	Pre‐post	To examine the role of screening expectations in modifying psychological responses to screening results among high‐risk individuals receiving LCS	Participants in UKLS who had completed LDCT *N* = 1589	T0 = Baseline T1 = 2 weeks after baseline LDCT scan result	Gender Age Deprivation Marital status Smoking status Experience of lung cancer Expectation result congruence	Concern about LDCT result	1 item	High
Eberth et al., 2022^32^	United States	Cross‐sectional	To explore how patients who have been referred for LCS by their healthcare provider describe the SDM visit, including what information they learned about screening and their level of certainty about their screening decision	Patients completing LDCT screening *N* = 75	Within 10 days of LDCT being ordered	Gender Age Race/ethnicity Education Employment Knowledge	Decisional conflict	Items adapted from the SURE scale	High
Golden et al., 2020^44^	United States	Qualitative	To use the LCS decision discussion as a case study to understand possible underlying components of a teachable moment to enhance motivation for smoking cessation	Patients who had completed SDM for LCS during routine care *N* = 51 Elected LDCT: *N* = 43 Declined LDCT: *N* = 8	After SDM and either before LDCT for electors, or within 3 weeks post‐SDM for decliners	Risk perception	Distress Motivation to quit	Semi‐structured interviews	Moderate
Golden et al., 2022^51^	United States	Qualitative	To (1) determine whether teachable moments for smoking cessation occur downstream from the initial LCS decision‐making interaction, (2) investigate patient experiences with smoking cessation and recommendations for improving cessation rates within LCS	Patients who had completed SDM for LCS during routine care *N* = 39 (61 interviews) T1 = 32 T2 = 29 Elected LDCT: *N* = 32 Declined LDCT: *N* = 7	T1 = 2–4 weeks post‐LDCT for LDCT electors; 4 weeks post‐SDM for LDCT decliners T2 = 12 months after SDM (regardless of LDCT decision)	Distress	Motivation to quit	Semi‐structured interviews	Moderate
Greene et al., 2019^40^	United States	Qualitative	To explore how those at highest risk for lung cancer (people who currently smoke) experienced, understood and made decisions about participation in LCS after being offered during routine primary care visits	Individuals who had been offered screening at a routine primary care visit *N* = 37 Elected LDCT: *N* = 33 Declined/delayed LDCT: *N* = 4	Within 4 weeks of first screening offer	Smoking history Experience with lung cancer	Fear Guilt, shame, self‐blame and regret	Semi‐structured interviews	Moderate
Hall et al., 2018^41^	United States	Cross‐sectional	To (1) quantify referral clarity and perceived accuracy during LCS; (2) identify medical, sociodemographic, smoking, behaviour and numeracy correlates to LCS uncertainty; (3) demonstrate associations between LCS uncertainty and emotional functioning	Patients who had recently completed LDCT *N* = 169	After LDCT (within a few weeks)	Education Numeracy (objective and subjective) Health insurance Smoking status Medical conditions Perceived accuracy of LCS	Perceived stress	PSS‐4	High
							Anxiety	GAD‐2	
							Referral clarity	1 item	
							Perceived accuracy of LCS	1 item	
Han et al., 2019^31^	United States	Mixed‐methods (pre‐post surveys for outcomes of interest)	To evaluate the effects of providing personalized cancer risk information (PCRI) to patients referred for LCS	Patients referred for LDCT who received PCRI *N* = 60	Immediately pre‐SDM Immediately post‐SDM	Gender Age Calculated lung cancer risk (using PLCOm2012)	Perceived lung cancer risk Perceived uncertainty about lung cancer risk Interest in quitting smoking	1 item per outcome Semi‐structured interviews	High
Kaerlev et al., 2012^26^	Denmark (DLCST)	Cross‐sectional	To examine psychological adverse effects in LCS participants with the calculation of risk ratios for prescription of anti‐depressive or anxiolytic medication	Participants randomised to an intervention group (CT scan) or control group in the DLCST *N* = 4104 Intervention: *N* = 2052 Control: *N* = 2052	Annual follow‐up over 3 years	Gender Age Civil status Socio‐economic status Comorbidities Prescription of antidepressant or axiolytic medication at least once during the 4‐month period before baseline	Use of antidepressant or axiolytic medication	Prescription of antidepressant or axiolytic medication	Moderate
Kathuria et al., 2020^48^	United States	Qualitative	To characterise perspectives of physicians and LCS participants on communication and perceived utility of LCS integrated with smoking cessation	Patients who underwent LCS in the previous year *N* = 28	Within 1 year after LCS and results	Perception of tobacco use on health	Motivation to quit	Semi‐structured interviews and focus groups	Moderate
Kummer et al., 2020a^17^	England (LSUT)	Qualitative	To explore the spectrum of psychological and behavioural responses among individuals with indeterminate and incidental LDCT results	Patients who had received a Lung Health Check as part of the LSUT trial *N* = 28	4–8 months (μ = 6 months) after LDCT as part of the Lung Health Check	Smoking status Existing concerns about lung health and smoking history Expectation of LDCT result Perceived stigma Fatalism Risk perception Social support	Psychological outcomes (anxiety, worry, relief, reassurance, guilt and emotional well‐being) Health beliefs (stigma, fatalism and affective risk perception) Motivation to quit Seeking social support	Semi‐structured interviews	Moderate
Kummer et al., 2020b^42^	England (LSUT)	Pre‐post	To (1) investigate sociodemographic and smoking‐related characteristics associated with psychological outcomes following LCS, and (2) compare psychological outcomes for screened vs. ‘screening unaware’ (community comparison sample)	Patients undergoing a LDCT pilot trial *N* = 787	T0 = LHC appointment T1 = next day T2 = 3‐month follow‐up	Gender Age Ethnicity Education Employment status Marital status Smoking status	Cancer worry	Adapted version of CWS	Moderate
							Anxiety	HADS	
							Depression	HADS	
Lebrett et al., 2022^22^	England (Manchester LHC)	Cross‐sectional	To examine lung cancer risk perception, disease knowledge and lung cancer‐specific worry in attendees of a community‐based LCS programme and explore association between these measures and lung cancer risk scores, screening eligibility and other variables	Individuals attending for a Lung Health Check who consented for linkage of responses with clinical data *N* = 243	Immediately prior to LHC	Gender Age Education Smoking status Pack years PLCO_m2012_ risk score Body Mass Index Previous cancer Family history of lung cancer Perceived risk (absolute and comparative) Anxiety and depression (PHQ‐4) Lung cancer worry (frequency)	Perceived risk (absolute and comparative)	2 items	Moderate
							Lung cancer worry (frequency and impact)	2 items adapted from CWS	
							Anxiety	PHQ‐4	
							Depression	PHQ‐4	
Lee et al., 2021^28^	Korea (K‐LUCAS)	Pre‐post	To report the interim results of baseline screening during the K‐LUCAS trial (to December 2017)	K‐LUCAS trial participants (people who currently or formerly smoke at high‐risk aged 55–74 years with an at least 30‐pack‐year smoking history) *N* = 5597	Day of screening After receiving results	Age Education Income Smoking status	Anxiety Motivation to quit	1 item per outcome	High
Lillie et al., 2017^39^	United States	Cross‐sectional	To (1) identify which factors people consider most important in making LCS decisions; (2) explore whether factors considered important vary by individual characteristics; (3) detect whether perceived importance of benefits and harms of screening varied by LCS completion	Veterans randomised to receive direct LCS invitation with decision aid or usual care *N* = 588 Intervention (direct LCS invitation): *N* = 384 Usual care (provider referral for LCS): *N* = 204	3 months post‐randomisation to intervention or usual care group	Education Income Smoking status Self‐reported health status	Lung cancer risk perception Fear of lung cancer Anxiety of waiting for LDCT results	1 item per outcome (measured as % of participants rating certain decision‐making factors as important)	High
Nishi et al., 2021^33^	United States	Cross‐sectional	To describe the quality of SDM among people who recently received LCS	Patients who had completed LDCT within 12 months *N* = 266	Within 12 months of last LDCT	Gender Age Race Education Smoking status Pack years Time since last screening Number of times screened for lung cancer	Decisional conflict	SURE scale (4‐item short version of DCS)	High
Olson et al., 2022^37^	Australia (ILST)	Qualitative	To examine participants' emotionally imbued experiences of LCS	Individuals who currently smoke undergoing LDCT as part of the ILST trial *N* = 27	Immediately post‐LDCT and prior to receiving results	Gender Disclosure of results to family	Stigma Guilt, shame	Semi‐structured interviews	Low
Ostroff et al., 2001^46^	United States (ELCAP)	Cross‐sectional	To (1) describe self‐reported changes in smoking behaviour following enrolment in a LCS programme and (2) examine potential predictors and covariates of change in smoking behaviour	Participants enrolled in an LCS programme for high‐risk people who smoke who had undergone baseline LDCT *N* = 134	After LCS	Perceived risk of lung cancer	Motivation to quit	Telephone survey interview	Moderate
Quaife et al., 2021^4^	United Kingdom (SUMMIT study)	Prospective cohort (cross‐sectional for outcomes of interest)	To test whether (and which) psychological factors are associated with screening uptake behaviour prospectively using a longitudinal cohort design embedded within a multicentre LCS implementation trial	Participants invited for a Lung Health Check *N* = 7730	One week after LHC invitation	Gender Age Ethnicity Deprivation level Relationship status Smoking status	Perception of consequences of lung cancer Perception of personal control Perception of treatment control Illness coherence Emotional representation/response (worry, anxiety and fear) Risk perception Perceived stigma Perceived survival from lung cancer (fatalism) Perceived efficacy of smoking cessation	SRQ‐LCS, 25	Low
Taghizadeh et al., 2019^25^	Canada (Pan‐Can study)	Pre‐post	To describe changes in anxiety and HRQoL in a high‐risk Canadian cohort undergoing LCS	Individuals undergoing LDCT as part of the PanCan study *N* = 1237 T0 = 1237 T1 = 953 T2 = 1066	T0 = study enrolment (baseline) T1 = within 1 month after the CT results (1 month post baseline) T3 = prior to first annual repeat LDCT (12 months post baseline)	Gender Age Smoking status Pack years Alcohol consumption Family history of cancer Concern about getting lung cancer	Anxiety	STAI	High
Turner et al., 2021^30^	Canada (Pan‐Can study)	Cross‐sectional	To clarify the determinants of lung cancer risk perception and its role in LCS programmes and recruitment	Participants undergoing an LDCT scan as part of the PanCan study *N* = 2514	Baseline questionnaire at time of LDCT	Sex Age Education level Smoking status Pack years Average cigarettes/day smoked Age started smoking Serious attempt to quit (of those who are presently a smoking) High‐risk occupation Symptoms Comorbidities Known COPD # of comorbities Prior cancer Family history of cancer Previous chest radiographs Previous CT scans HRQoL (SF‐12 score) PLCO_m2012_ risk score Lung cancer risk perception Lung cancer worry	Lung cancer worry Risk perception Intention to quit smoking	1 item per outcome	Moderate
Van den Bergh et al., 2008^24^	Netherlands (NELSON)	Pre‐post	To assess discomfort experienced by subjects during LDCT and while waiting for results and explore the impact of LCS on HRQoL over time	Participants in the NELSON trial who received a negative or indeterminate baseline LDCT result (positive results excluded from analysis) *N* = 324 (returned T1 questionnaire) *N* = 270 (returned all questionnaires)	T1 = 1 week before baseline LDCT T2 = 1 day after baseline LDCT (no results received) T3 = 6 months after baseline LDCT (post‐results)	Gender Age Smoking status Previous CT scan	Anxiety	STAI‐6	Moderate
							Lung cancer‐specific distress	IES	
							HRQoL	EQ‐5D SF‐12	
							Discomfort experienced during LDCT	Multiple items	
Van den Bergh et al., 2010a^36^	Netherlands/Belgium (NELSON)	Pre‐post	To assess changes in generic and lung cancer‐specific HRQoL changes over time among participants undergoing LCS in the short term	Patients in the NELSON trial who received a negative or indeterminate baseline LDCT result (positive results excluded from analysis) *N* = 630 (returned T0 questionnaire) *N* = 494 (returned all questionnaires)	T0 = Baseline (~5 months before LDCT) T1 = ~2 days before LDCT T2 = ~4 days after baseline scan (no results received) T3 = After results (3 months after baseline scan; ~3 months before follow‐up scan for indeterminate results)	Gender Smoking status Pack years	Anxiety	STAI‐6	High
							Lung cancer‐specific distress	IES	
							HRQoL	EQ‐5D SF‐12 (T0, T3 only)	
Van den Bergh et al., 2010b^50^	Netherlands/Belgium (NELSON)	Pre‐post	To evaluate whether LCS participants who made an informed decision had better HRQoL than those who did not make an informed decision, especially those receiving an indeterminate test result which required a follow‐up CT scan	Patients in the NELSON trial who made or made not an informed decision to participate in LCS *N* = 288 Informed decision: *N* = 155 No informed decision: *N* = 133	T0 = Baseline T1 = 1 week before baseline scan T2 = 1 day after baseline scan	Informed decision (defined as having adequate knowledge, positive attitude and participation in LCS)	Anxiety	STAI‐6	High
							Lung cancer‐specific distress	IES	
							HRQoL	EQ‐5D SF‐12	
							Psychological consequences of LCS (T2 only)	Part 1 of COS‐LC	
							Risk perception (cognitive)	1 item	
							Risk perception (affective)	1 item	
Van den Bergh et al., 2011^18^	Netherlands/Belgium (NELSON)	Pre‐post	To 1) compare HRQoL in a LCS and control group over 2 years; 2) explore the short‐term effects on HRQoL of an indeterminate result at second‐round screening; 3) evaluate the long‐term effects of an indeterminate baseline result; and 4) evaluate the differences between getting a negative follow‐up scan and getting at least one indeterminate or positive result at follow‐up	Patients in the NELSON trial, randomised to either receive LCS or control. *N* = 1288 (returned T0 questionnaire) LCS: *N* = 658 Control: *N* = 630	T0 = Baseline (~5 months before LDCT) T1 = After results (~1 month after baseline result; ~3 months before follow‐up scan for indeterminate results) T2 = ~1.5 years after baseline scan (~6 months after second LDCT for those eligible)	Gender Age Education Smoking status Pack years	Anxiety	STAI‐6	Moderate
							Lung cancer‐specific distress	IES	
							HRQoL	EQ‐5D SF‐12	
Williams et al., 2022^35^	USA (LSTH)	Pre‐post	To (1) examine changes in readiness to quit, motivation to quit, and cigarettes per day from before screening to after the receipt of lung screening results, and (2) examine the extent to which the teachable moment domains of perceived risk for lung cancer and lung cancer worry were associated with changes in these smoking‐related attitudes and behaviours	Patients completing baseline or annual LDCT scan *N* = 843	T0 = Baseline (before LDCT) T1 = 1‐week post‐scan (after receipt of results)	Sex Age Race Smoking characteristics (pack‐years, smoking habits, quit attempts and cigarettes per day) LDCT history Perceived comparative risk Perception of health Lung cancer worry	Readiness to quit	Contemplation Ladder	Moderate
							Motivation to quit	1 item	
Zeliadt et al., 2015^47^	United States	Qualitative	To understand views on smoking cessation from people who currently smoke in the context of being offered LCS as a routine service in primary care	Patients who had been offered LCS as part of a LCS Clinical Demonstration Project *N* = 37	T0 = after offer of LDCT, before actual LDCT (*N* = 7) T1 = after screening and results (*N* = 22) (8 interviewed at both T0 and T1)	Expectation of results	Relief	Interviews	High

Abbreviations: CES‐D, Center for Epidemiologic Studies Depression Scale; CI, confidence interval; COS‐LC, Consequences of Screening in Lung Cancer questionnaire; CWS, Cancer Worry Scale; DCS, decisional conflict scale; DLCST, Danish Lung Cancer Screening Trial; ECLS, Early Diagnosis of Lung Cancer Scotland trial; ELCAP, Early Lung Cancer Action Project; EQ‐5D, EuroQoL 5 dimension health‐related quality of life measure; GAD‐2, Generalised Anxiety Disorder‐2 scale; HADS, Hospital Anxiety and Depression Scale; HRQoL, health‐related quality of life; IES, Impact of Events Scale; IQR, interquartile range; K‐LUCAS, Korean Lung Cancer Screening Project; LCS, lung cancer screening; LDCT, low‐dose CT scan; LHC, Lung Health Check; LSTH, Lung Screening, Tobacco, and Health trial; LSUT, Lung Screen Uptake Trial; LUSI, German Lung Cancer Screening Intervention trial; MD, mean difference; MID, minimal important difference; NELSON, Dutch–Belgian Randomised Controlled Lung Cancer Screening Trial; OR, odds ratio; PanCan, Pan‐Canadian Early Detection of Lung Cancer Study; PCQ, Psychological Consequences Questionnaire; PANAS, Positive and Negative Affect Schedule; PCRI, personalised cancer risk information; PHQ‐4, Patient Health Questionnaire‐4; PLuSS, Pittsburgh Lung Screening Study; PSS‐4, 4‐item Perceived Stress Scale; RR, relative risk; SDM, shared decision‐making; SF‐12, 12‐Item Short Form Health Survey; SRQ‐LCS 25, self‐regulatory questionnaire for lung cancer screening; STAI, State–Trait Anxiety Inventory; UKLS, United Kingdom Lung Cancer Screening Trial.

Risk of bias was assessed as high (*n* = 16), moderate (*n* = 17) and low (*n* = 2). Quality ratings for quantitative studies included in this review were commonly hindered by possible selection bias and coverage bias, and sample sizes were also often small and/or unjustified. Quasi‐experimental study designs frequently lacked control groups, and cross‐sectional designs had low response rates. Full quality appraisals according to the JBI critical appraisal tool checklists are provided in Tables S3–S5.

A summary of the factor–outcome combinations examined is provided in Table S2. Key results for each factor–outcome combination are summarised in Table 2 and are presented narratively below by broad outcome categories: psychological outcomes (both general and lung cancer or LCS‐specific; e.g. anxiety and lung cancer‐specific worry), beliefs (e.g. risk perception and fatalism), decision‐related (e.g. decisional conflict), and smoking‐related (e.g. motivation to quit smoking).

**TABLE 2:** Summary of key results.

Factor (# of studies)	Outcome of interest	Results
*Sociodemographic factors*		
Age (*n* = 19)	Psychological (*n* = 12)	Younger age was associated with worse psychological outcomes in seven studies.^17^, ^18^, ^19^, ^20^, ^21^, ^22^, ^23^ The most commonly found association was with lung cancer or LDCT‐specific cancer distress or concern (*n* = 4).^18^, ^19^, ^20^, ^21^ However, most (*n* = 9) analyses found no significant differences in psychological outcomes by age,^17^, ^18^, ^19^, ^22^, ^24^, ^25^, ^26^, ^27^, ^28^ including for anxiety^18^, ^22^, ^25^, ^28^ (*n* = 4) HRQoL^18^, ^24^ (*n* = 2), cancer worry^17^, ^22^ (*n* = 2) and fear of lung cancer^19^, ^27^ (*n* = 2)
	Beliefs (*n* = 7)	Younger age was associated with higher perceived lung cancer risk in three studies,^21^, ^29^, ^30^ but not in three others.^19^, ^22^, ^31^ In a high‐quality cross‐sectional study, older age was associated with more perceived control over lung cancer treatment, more positive perception of consequences of lung cancer, and less perceived stigma.^21^ There was no significant association between age and the importance of perceived efficacy of LCS for making a LCS decision in one study^27^
	Decision‐related (*n* = 3)	Associations between age and decisional outcomes was measured in three studies;^27^, ^32^, ^33^ two studies found no significant difference in decisional conflict by age^32^, ^33^
	Smoking‐related (*n* = 5)	Age and smoking‐related psychosocial outcomes evidence was mixed, with most studies (*n* = 3) reporting no significant relationship between age and interest, readiness, motivation or concerns about quitting smoking.^31^, ^34^, ^35^ One study reported that increasing age was significantly associated with less perceived benefit of smoking cessation.^21^
Gender (*n* = 20)	Psychological (*n* = 12)	Across 10 studies,^17^, ^18^, ^19^, ^20^, ^21^, ^22^, ^24^, ^25^, ^26^, ^36^ women were consistently reported to have worse psychological outcomes, including cancer‐related distress, fear or worry^17^, ^18^, ^19^, ^20^, ^21^, ^22^, ^36^ (*n* = 7) and anxiety^17^, ^18^, ^22^, ^25^, ^36^ (*n* = 5). However, these findings were not always clinically meaningful. Six studies^17^, ^19^, ^22^, ^23^, ^24^, ^27^ found no significant relationship between psychological outcomes and gender, including for cancer‐related distress, fear or worry^17^, ^23^, ^24^, ^27^ (*n* = 4), depression^17^, ^22^ (*n* = 2) and anxiety^19^, ^24^ (*n* = 2).
	Beliefs (*n* = 8)	Some analyses (*n* = 3) reported women to have higher perceived risk of lung cancer;^21^, ^22^, ^30^ while others (*n* = 4) reported no difference by gender.^19^, ^22^, ^29^, ^31^ In a high‐quality cross‐sectional study, women were reported to have less feelings of personal and treatment control.^21^ Two studies (one qualitative and one quantitative) each reported stigma was more common in women.^21^, ^37^ Women rated the perceived efficacy of LCS as significantly more important for making a LCS decision in one study.^27^
	Decision‐related (*n* = 3)	Associations between gender and decisional outcomes was measured in three studies.^27^, ^32^, ^33^ Two studies found no difference in decisional conflict by gender,^32^, ^33^ one study reported women having significantly lower decisional regret than men.^27^
	Smoking‐related (*n* = 4)	Studies mostly reported no significant association between gender‐ and smoking‐related psychosocial outcomes (*n* = 4),^21^, ^31^, ^34^, ^35^ except women being more likely to endorse concerns about weight gain after quitting in one study.^34^
Race/ethnicity (*n* = 9)	Psychological (*n* = 5)	Four studies reported no differences in psychological outcomes across race or ethnicity,^17^, ^19^, ^20^, ^38^ primarily for lung cancer or LDCT distress or fear. Two others reported worse outcomes for participants with African American^38^ (*n* = 1) or Asian^21^ (*n* = 1) ethnic backgrounds.
	Beliefs (*n* = 3)	Three studies measured risk perception and found no significant relationship with race.^19^, ^21^, ^38^ Higher fatalism and more negative perceptions of personal control were reported for participants with African American^38^ or Asian^21^ ethnic backgrounds in one study each. Though in one study, both participants with Black and Asian ethnic backgrounds had significantly more positive perceptions of the consequences of lung cancer and perceptions of treatment control, while White participants reported higher perceived stigma.^21^
	Decision‐related (*n* = 2)	Across two studies, White^32^, ^33^ (*n* = 2) and Hispanic^33^ (*n* = 1) participants were significantly less likely to experience decisional conflict about LCS than other ethnicities.
	Smoking‐related (*n* = 3)	There was no significant relationship between race/ethnicity and motivation to quit smoking^31^, ^34^, ^35^ (*n* = 2), readiness to quit smoking^35^ (*n* = 1) or perceived efficacy of smoking cessation^21^ (*n* = 1). In one study, those who identified as non‐Hispanic Black had significantly more concerns about withdrawal symptoms and were more likely to believe that they do not need to make smoking changes.^34^
Education (*n* = 11)	Psychological (*n* = 7)	Higher levels of education were associated with better psychological outcomes in four studies,^17^, ^18^, ^19^, ^22^ including for cancer‐related distress or fear^18^, ^19^, ^22^ (*n* = 3) and anxiety^18^, ^19^ (*n* = 2). However, analyses in another four studies^17^, ^20^, ^22^, ^39^ reported no relationship, specifically with cancer distress, fear, worry or impact on mood (*n* = 4)^17^, ^20^, ^22^, ^39^ and anxiety (*n* = 4).^17^, ^22^, ^39^ Results from the K‐LUCAS trial was the only study to show minimally higher anxiety in those with higher education levels, though this study had a high risk of bias and significance between groups was not calculated.^28^
	Beliefs (*n* = 5)	The association between risk perception and education was varied, with two studies reporting no relationship,^22^, ^39^ and one study reporting participants with higher education having lower perception of lung cancer risk.^19^ Higher objective numeracy scores, but not subjective numeracy or education level, were associated with the importance of perceived efficacy of LCS for making LCS decisions in one study.^27^
	Decision‐related (*n* = 2)	Two studies found no significant relationship between education level and decisional conflict about LCS.^32^, ^33^
	Smoking‐related (*n* = 1)	The K‐LUCAS trial reported higher motivation to quit smoking in the higher education group, however between‐group analysis was not completed.^28^
Income, employment, insurance and deprivation (*n* = 9)	Psychological (*n* = 7)	Seven studies examined the relationship between income, insurance, deprivation, and employment status with psychological outcomes. Five studies^17^, ^20^, ^23^, ^26^, ^28^ found worse psychological outcomes for those who had higher levels of deprivation, lower income or were unemployed; however, relationships in two of these studies were no longer significant after multivariate analysis.^17^, ^20^ In addition, three studies^17^, ^21^, ^39^ reported no significant association between psychological outcomes, with one of these being the only high‐quality (low risk of bias) quantitative study included.^21^
	Beliefs (*n* = 3)	Two studies found no significant relationship between income or deprivation level, and risk perception.^21^, ^39^ One high quality study reported that greater affluence was significantly associated with more negative perceptions of consequences and higher perceived stigma, but more positive perceptions of personal control.^21^ There was no association between fatalism and perception of treatment control.^21^ There was no significant association between insurance and the importance of perceived efficacy of LCS for making a LCS decision in one study.^27^
	Decision‐related (*n* = 1)	One study examined decisional conflict and employment, finding no significant association.^32^
	Smoking‐related (*n* = 2)	Greater affluence was significantly associated with lower perceived efficacy of smoking cessation in a high‐quality cross‐sectional study.^21^ The K‐LUCAS trial reported slightly higher motivation to quit smoking for those with lower income; however, between‐group analysis was not completed.^28^
Relationship status (*n* = 6)	Psychological (*n* = 6)	Married or co‐habiting participants were reported to have significantly better psychological outcomes in four studies,^17^, ^19^, ^20^, ^26^ however analyses across another four studies reported no association between relationship status and psychological burden,^17^, ^19^, ^21^, ^23^ with one of these being the only high‐quality (low risk of bias) quantitative study included.^21^
	Beliefs (*n* = 2)	Two studies reported no significant relationship between relationship or marital status and risk perception.^19^, ^21^ In one high quality cross‐sectional study, those who were in a partnership or co‐habiting had more positive perceptions of treatment control and fatalism, but there was no relationship with the consequences of lung cancer, perceptions of personal control or stigma.^21^
	Smoking‐related (*n* = 1)	One study found no significant relationship between perceived efficacy of smoking cessation and relationship status.^21^
*Health‐related factors*		
Health status (*n* = 5)	Psychological (*n* = 3)	Alcohol consumption^25^ and Body Mass Index (BMI)^22^ were not significantly associated with psychological outcomes across one study each. In another study, a participant's number of comorbidities was reportedly associated with antidepressive or anxiolytic use.^26^
	Beliefs (*n* = 3)	Having asthma,^30^ COPD^30^ and symptoms (such as dyspnoea or cough),^30^ but not BMI^22^ or the number of comorbidities,^30^ were associated with lung cancer risk perception in one study each. Medical conditions were unrelated to the importance of perceived efficacy of LCS for making LCS decisions in one study.^27^
Experience with cancer, lung cancer or LCS (*n* = 9)	Psychological (*n* = 6)	Personal or family experience with cancer (including lung cancer) was associated with higher lung cancer distress, concern about LDCT result or fear in three studies,^20^, ^23^, ^40^ but had no links to psychological burden in three other studies.^22^, ^24^, ^25^
	Beliefs (*n* = 2)	Family history of lung cancer was associated with comparative risk perception in two studies,^22^, ^30^ but not absolute risk perception in one.^22^ Previous cancer was not associated with risk perception in two studies.^22^, ^30^ One study reported an associated between the number of previous chest radiographs, but not CT scans, with risk perception.^30^
	Decision‐related (*n* = 1)	The number of times someone was previously screened for lung cancer, or the time since previous scan, were not related to decisional outcomes in one study.^33^
	Smoking‐related (*n* = 1)	In one study, those attending for a baseline scan (rather than an annual follow‐up scan) reported higher readiness and motivation to quit smoking.^35^
Smoking status and history (*n* = 21)	Psychological (*n* = 16)	Most analyses (*n* = 9) reported no significant association between smoking status (i.e. current versus former smoking) and outcomes such as anxiety^17^, ^18^, ^22^, ^25^, ^39^ (*n* = 5) and lung cancer‐specific fear, worry or concern (*n* = 4).^17^, ^21^, ^23^, ^27^ Seven studies reported worse psychological outcomes for participants who reported current smoking,^18^, ^19^, ^20^, ^22^, ^36^, ^39^, ^41^ though these was not always clinically important. Four studies reported that more pack years were associated with increased psychological burden,^18^, ^22^, ^28^, ^36^ but three studies also reported no significant relationship.^18^, ^22^, ^25^ In two qualitative studies, more pronounced smoking history was often linked to increased worry, guilt and shame in the context of LCS.^40^, ^42^
	Beliefs (*n* = 10)	The relationship between smoking status and risk perception was varied, reported as worse for people currently smoking (*n* = 4),^19^, ^21^, ^22^, ^39^ worse for people who had quit smoking (*n* = 1),^30^ and not significantly different (*n* = 2).^22^, ^29^ Higher number of pack years was associated with higher perceived risk in one study,^30^ but not in two others.^22^, ^30^ Younger smoking start age and higher average cigarettes smoked per day were also associated with higher risk perception in one study.^30^ Stigma was described by people currently smoking in two qualitative studies,^40^, ^42^ with significantly higher perceived stigma reported by this group in another quantitative study.^21^ Higher fatalism and reduced perceptions of control were also higher in those who were currently smoking.^21^ Two studies indicated no difference in perceived efficacy or accuracy of LCS by smoking status.^27^, ^41^
	Decision‐related (*n* = 2)	People who formerly smoked were more likely to report worse decisional outcomes compared with those currently smoking in two studies,^27^, ^33^ though a significant relationship was only reported in one.^33^ Pack years was not significantly associated with decisional conflict in one study.^33^
	Smoking‐related (*n* = 5)	Findings on the impact of smoking status on smoking‐related psychosocial outcomes were mixed. Current smoking status was associated with more smoking worry^43^ (*n* = 1) and a higher intention to quit^43^ (*n* = 1), but lower confidence in quitting^43^ (*n* = 1) and lower perceived efficacy of smoking cessation^21^ (*n* = 1), in conjunction with LCS. Another study reported that participants with greater nicotine dependence were significantly more likely to endorse concerns about quitting smoking.^34^ There was no difference in motivation to quit by pack years in two studies,^28^, ^35^ though readiness and motivation to quit was associated with being less likely to smoke every day and having made a 24‐hour quit attempt in the last 7 days in one study.^35^
Pre‐ or co‐existing psychological burden (*n* = 9)	Psychological (*n* = 5)	One study reported that for those with low distress at baseline, LDCT participants (versus non‐participants) had significantly higher distress 2 weeks post‐results.^20^ However, for those with initial high distress, completing LDCT was not impactful, and overall distress remained high.^20^ Pre‐existing concern about lung cancer was associated with anxiety about LCS (and consequential relief following LDCT results) in one qualitative study.^42^ Three studies indicated that pre‐existing levels of anxiety and depression were associated with worse harm in other psychological outcomes at different points during LCS.^22^, ^25^, ^26^
	Beliefs (*n* = 2)	Two studies reported a significant relationship between worse psychological outcomes (anxiety, depression and HRQoL) and higher absolute and comparative risk perception.^22^, ^30^ However, one study also reported no association between lung cancer worry and risk perception.^22^
	Smoking‐related (*n* = 5)	Worry or concern about lung cancer was significantly associated with readiness or motivation to quit smoking in two quantitative studies.^30^, ^35^ This relationship was qualitatively described in three studies;^42^, ^44^, ^45^ though, the reported association was more varied. Psychological harm experienced during LCS appeared to motivate quitting smoking for some participants, while it had no effect for others^42^, ^44^, ^45^
Calculated risk of lung cancer (*n* = 3)	Psychological (*n* = 2)	Calculated lung cancer risk had varying impacts on psychological outcomes across two studies, but primarily results indicated no significant relationship.^22^, ^30^
	Beliefs (*n* = 3)	Calculated lung cancer risk via PLCO_m2012_ was not associated with absolute or comparative risk in two studies,^22^, ^31^ but was associated with comparative risk in one study.^30^
*Beliefs*		
Lung cancer risk perception (*n* = 7)	Psychological (*n* = 4)	Two quantitative studies found that both comparative risk perception and affective risk perception (feelings about risk) resulted in more psychological burden.^22^, ^29^ Two qualitative studies, however, suggested that even though participants sometimes overestimated their risk of lung cancer, this did not cause distress (but noted that there were differences among participants).^42^, ^44^
	Beliefs (*n* = 1)	The only study looking at the relationships between absolute and comparative risk reported a significant association, where participants with higher perceived absolute risk were more likely to also perceive themselves to be at ‘higher’ comparative risk.^22^
	Smoking‐related (*n* = 4)	Two studies reported that those with higher perceived risk showed more motivation or intention to quit smoking (*n* = 2),^30^, ^46^ though one study suggested the inverse^35^ and two studies suggested no relationship between risk perception and motivation to quit.^35^, ^44^
Expectation of LDCT result (*n* = 4)	Psychological (*n* = 3)	One study specifically measured how LDCT result expectations impacted psychological outcomes during LCS.^23^ Participants who expected and received a negative (‘normal’) result had significantly lower concern than any other result expectation group 2 weeks after receiving results.^23^ There was no significant difference in concern for those with an unexpected versus an expected ‘positive’ scan (i.e. requiring follow‐up).^23^ Across qualitative studies, participants who received an unexpected abnormal result reported more psychological burden,^42^ participants who received an unexpected ‘negative’ result felt relief and reduced stress^47^ and being ‘psychologically prepared’ for a possible indeterminate result appeared to provide an emotional buffer.^42^
	Smoking‐related (*n* = 1)	One qualitative study reported that having an unexpected ‘negative’ scan reportedly made participants ‘feel it was worth making a change’ and provided motivation to quit smoking.^45^
Perception of health (*n* = 3)	Psychological (*n* = 1)	Lower perception of health was non‐significantly associated with fear of lung cancer, and significantly associated with anxiety of waiting for LDCT results in one study.^39^
	Beliefs (*n* = 1)	There was no association between perception of health and lung cancer risk perception in one study.^39^
	Smoking‐related (*n* = 2)	Health perception was only significantly associated with motivation to quit in one study, but not readiness to quit.^35^ In a qualitative study, those who downplayed the impact of tobacco use on health appeared to have lower motivation to quit smoking in the context of LCS.^48^
Fatalism (*n* = 1)	Psychological (*n* = 1)	Qualitatively in one study, higher fatalism was reported to result in relief and other positive psychological responses following any type of LDCT result.^42^
	Social (*n* = 1)	One qualitative study suggested that having a fatalistic outlook reduced seeking of social support.^42^
Stigma (*n* = 1)	Psychological (*n* = 1)	Stigma from smoking appeared to drive higher worry and lack of reassurance from a ‘normal’ LDCT scan result in one qualitative study.^42^
	Beliefs (*n* = 1)	A qualitative study reported a relationship between perceived stigma and fatalism, indicating that some participants felt ‘lucky’, while others felt that it was ‘only a matter of time’.^42^
Perception of LCS (*n* = 1)	Psychological (*n* = 1)	One study found that perceived accuracy of LCS was not associated with perceived stress or anxiety.^41^
*Other factors*		
Individual responses to COVID‐19 (*n* = 2)	Smoking‐related (*n* = 2)	Two qualitative studies described the pandemic as creating additional stress and anxiety that reduced motivation or readiness to quit smoking,^45^, ^49^ this was reported as both specific to smoking cessation interventions during LCS^45^ and more generally.^49^ However, for a minority, COVID‐19 increased motivation as people were more conscious of their smoking habits and could make changing smoking behaviour a priority.^49^
Informed decision‐making and knowledge (*n* = 2)	Psychological (*n* = 1)	One study reported that participants who did not make an informed decision to participate in LCS experienced no worse anxiety, HRQoL or lung cancer specific distress than subjects who did make an informed decision (including after receiving an indeterminate result).^50^
	Beliefs (*n* = 1)	One study examined the impact of informed decision making on risk perception, finding that those with an informed decision had more accurate cognitive perceptions of risk, though there was no relationship with affective risk perception^50^
	Decision‐related (*n* = 1)	One study found a significant correlation between knowledge and decisional conflict.^32^
Social factors (*n* = 2)	Psychological (*n* = 2)	Two qualitative studies reported on social factors. One suggested that social support provided an important ‘buffer’ of emotional support throughout the screening pathway.^42^ Another suggested that having to disclose results to family members resulted in guilt and shame (possibly driven by internalised blame or stigma around smoking).^37^

### 3.2 Psychological outcomes

Twenty‐four studies measured psychological outcomes. Across 10 studies, women were reported to have worse psychological outcomes than men during LCS, but these were not always clinically meaningful,^17^, ^18^, ^19^, ^20^, ^21^, ^22^, ^24^, ^25^, ^26^, ^36^ and some analyses (*n* = 6) reported no significant relationship with gender.^17^, ^19^, ^22^, ^23^, ^24^, ^27^ Younger age was associated with worse psychological outcomes in seven studies,^17^, ^18^, ^19^, ^20^, ^21^, ^22^, ^23^ though most analyses (*n* = 9) found no significant differences by age.^17^, ^18^, ^19^, ^22^, ^24^, ^25^, ^26^, ^27^, ^28^ Better psychological outcomes were found across four studies for those with higher levels of education^17^, ^18^, ^19^, ^22^ and married and co‐habiting participants,^17^, ^19^, ^20^, ^26^ though non‐significant associations were also reported in four studies for each factor.^17^, ^19^, ^20^, ^21^, ^22^, ^23^, ^39^ Four studies found no differences in outcomes across race or ethnicity,^17^, ^19^, ^20^, ^38^ while worse outcomes for participants from African American^38^ or Asian^21^ ethnic backgrounds were reported in one study each.

Across 16 studies examining associations between smoking characteristics and psychological outcomes, seven studies reported worse outcomes for people who were currently smoking,^18^, ^19^, ^20^, ^22^, ^36^, ^39^, ^41^ while nine studies reported no significant association with smoking status.^17^, ^18^, ^21^, ^22^, ^23^, ^24^, ^25^, ^27^, ^39^ Similarly, higher pack years and a more pronounced smoking history were linked to increased psychological burden in some cases (*n* = 6),^18^, ^22^, ^28^, ^36^, ^40^, ^42^ but not others (*n* = 3).^18^, ^22^, ^25^ Personal or family experience with cancer (including lung cancer) was significantly associated with worse psychological outcomes in half of the studies which examined the factor (*n* = 3).^20^, ^23^, ^40^ Five studies reported the impact of pre‐existing or comorbid psychological burden, all of which indicated an association with psychological harm during LCS.^20^, ^22^, ^25^, ^26^, ^42^ One study also suggested that for those with low distress at baseline, LDCT participants had higher distress than non‐participants after receiving results.^20^

Findings on the impact of risk perception on psychological outcomes were mixed and appear to be individualised (*n* = 4).^22^, ^29^, ^42^, ^44^ Expectation of LDCT results impacted subsequent psychological outcomes differently across three studies.^23^, ^42^, ^47^ One study suggested preparation for an ‘indeterminate’ or ‘abnormal’ LDCT result could reduce psychological burden,^42^ but this had no reported effect in another study.^23^ Those who received an expected ‘normal’ result reported the lowest concern in one study;^23^ another described that if a ‘normal’ result was unexpected then this also led to relief and reduced stress.^47^ The impact of perceived stigma and fatalistic health beliefs were examined in one qualitative study; while higher fatalism led to positive psychological outcomes after receiving results (relief and reassurance), smoking‐related stigma appeared to have the opposite effect.^42^

One study reported that making an informed decision to participate in LCS had no impact on psychological outcomes, including after receiving an indeterminate result.^50^ Two qualitative studies examined social factors, with one suggesting that social support provided a ‘buffer’ of emotional support throughout the LCS pathway,^42^ and the other describing feelings of guilt and shame in having to inform family members (possibly driven by internalised blame or stigma around smoking).^37^

### 3.3 Beliefs

#### 3.3.1 Lung cancer risk perception

Risk perception was measured as an outcome in nine studies. Higher perceived lung cancer risk was associated with younger age^21^, ^29^, ^30^ and female gender^21^, ^22^, ^30^ in three studies each, though no relationship was reported in three^19^, ^22^, ^31^ and four^19^, ^22^, ^29^, ^31^ studies, respectively. No association was reported between risk perception and race or ethnicity^19^, ^21^, ^38^ (*n* = 3), income or deprivation level^21^, ^39^ (*n* = 2) or relationship status^19^, ^21^ (*n* = 2). Most studies reported no relationship with education level^22^, ^39^ (*n* = 2) as well, though one study reported those with higher education having lower risk perception.^19^ Family history of lung cancer was associated with comparative risk perception (risk compared to others) in two studies,^22^, ^30^ but not absolute risk perception in one study.^22^ Previous cancer was also not associated with risk perception in two studies.^22^, ^30^ The association between smoking status and risk perception varied, reported as worse for those currently smoking (*n* = 4),^19^, ^21^, ^22^, ^39^ those who had quit smoking (*n* = 1)^30^ and not significantly different (*n* = 2).^22^, ^29^ Higher number of pack years was associated with higher perceived risk in one study,^30^ but not in two others.^22^, ^29^

Two studies reported a significant relationship between increased risk perception and worse psychological outcomes (anxiety, depression and HRQoL),^22^, ^30^ but not lung cancer worry.^22^ Calculated lung cancer risk via PLCO_m2012_ was not associated with risk perception in two studies,^22^, ^31^ but was in another.^30^ One study examined the impact of informed decision‐making on risk perception, finding that those with an informed decision had more accurate cognitive perceptions of risk, though there was no relationship with perceived affective (feelings about) this risk.^50^

#### 3.3.2 Fatalism and perceptions of control

Three studies measured health beliefs related to fatalism and perceptions of control. Higher fatalism was linked to not being in a relationship or co‐habiting (*n* = 1),^21^ being of African American (*n* = 1)^38^ or Asian (*n* = 1)^21^ ethnic backgrounds, and current smoking status (*n* = 1).^21^ One high‐quality cross‐sectional study specifically measured other health beliefs, finding that more negative perceptions of personal control were associated with female gender, less affluence, current smoking status and Asian ethnicity.^21^ The same study found that participants with Black and Asian ethnic backgrounds had more positive perceptions of the efficacy of lung cancer treatment, as did men, those who were older, and those who were married, co‐habiting or in a partnership.^21^ Differences in perceptions of stigma were described in one qualitative study as moderating fatalistic outlooks.^42^

#### 3.3.3 Stigma

Four studies examined stigma, with two reporting that perceived stigma was more pronounced for women.^21^, ^37^ Younger age and White ethnicity were also associated with higher perceived stigma in one study.^21^ Stigma related to current smoking status was described in two qualitative studies.^40^, ^42^ Further, significantly higher perceived stigma was reported by people who were currently smoking in another quantitative study.^21^

### 3.4 Decision‐related psychosocial outcomes

Three studies measured decision‐related psychosocial outcomes. Most reported no significant difference in outcomes for age (*n* = 2),^32^, ^33^ level of education (*n* = 2),^32^, ^33^ employment status (*n* = 1)^32^ or gender (*n* = 2).^32^, ^33^ People who had quit smoking were more likely to report worse decisional outcomes compared with people who were currently smoking in two studies,^27^, ^33^ though a significant relationship was only reported in one.^33^ White^32^, ^33^ (*n* = 2) and Hispanic^33^ (*n* = 1) participants were reported as being less likely to experience decisional conflict about LCS than other ethnicities. One study reported that the number of times screened for lung cancer, or time since last scan, was not related to decisional outcomes.^32^

### 3.5 Smoking‐related psychosocial outcomes

Smoking‐related psychosocial outcomes were assessed in 14 studies. Most analyses reported no significant relationship between smoking‐related outcomes and age (*n* = 3),^31^, ^34^, ^35^ gender (*n* = 4)^21^, ^31^, ^34^, ^35^ or race/ethnicity (*n* = 3),^21^, ^34^, ^35^ except women being more likely to endorse concerns about weight gain after quitting and African American participants being less likely to endorse concerns about nicotine withdrawal symptoms in one study.^34^ Current smoking status was associated with more smoking worry^43^ (*n* = 1) and a higher intention to quit^43^ (*n* = 1), but lower confidence in quitting^43^ (*n* = 1) and lower perceived efficacy of smoking cessation^21^ (*n* = 1), in conjunction with LCS. There was no difference in motivation to quit by pack years in two studies.^28^, ^35^

Worry or concern about lung cancer was associated with readiness or motivation to quit smoking in five studies,^30^, ^35^, ^42^, ^45^, ^51^ though with varying impacts. For some participants, psychological harm experienced during LCS appeared to motivate quitting smoking,^30^, ^35^, ^45^ while it had no (or the opposite) effect for others.^42^, ^44^, ^45^ Two studies reported that those with higher perceived risk showed more motivation or intention to quit smoking (*n* = 2),^30^, ^46^ though one study suggested the inverse^35^ and another two studies suggested no relationship between risk perception and motivation to quit.^35^, ^44^ Two qualitative studies described the COVID‐19 pandemic as creating additional stress and anxiety that reduced motivation or readiness to quit smoking;^45^, ^49^ this was reported as both specific to smoking cessation interventions during LCS^45^ and more generally.^49^

### 3.6 Social outcomes

Social outcomes were only described in one qualitative study, which reported that a fatalistic outlook reduced seeking of social support.^42^

## 4 DISCUSSION

This review found that several participant factors were consistently reported to be associated with psychosocial outcomes in LCS. However, associations were not consistent across studies and non‐significant findings also frequently reported. This may be due to bias in study designs and lack of power to show associations; indeed, small and/or unjustified sample sizes were an issue across many quantitative studies in risk of bias assessment. Many of the factors and outcomes examined were also interrelated, which should be considered in interpretation of findings. Sociodemographic and smoking‐related characteristics were the most frequently examined factors. Correlations between younger age, female gender, and current smoking status, and increased psychological burden were commonly reported. Similar findings were reported recently in the Watch the Spot trial (which included those with both screening and incidentally detected nodules).^52^ Links with pre‐existing or comorbid psychological harm, expectation of LDCT results and other beliefs were also found, though were examined in fewer studies and were more nuanced. While this reviews' results are limited by study quality (as systematically assessed in our work), they provide a basis for understanding who may be at risk of psychological harm during LCS based on all available evidence. These findings are also useful when considering psychological barriers to LCS uptake and developing campaigns or strategies to engage certain groups.^5^

Considering the evidence for risk factors associated with psychological harm found in this review, interventions to manage psychosocial experiences during LCS should not be ‘one size fits all’. Different assessment, referral and intervention pathways which target specific factors or at‐risk groups should be considered in LCS programme design. For example, people who live alone or do not have support systems could be assessed and referred to an intervention focused at providing social support, which have been shown to improve quality of life for lung cancer patients.^53^ Acknowledging that most participants in LCS do not experience clinically significant psychological burden, avoiding a blanket universal intervention approach may also have cost‐effectiveness benefits,^54^, ^55^ especially considering participation volumes in larger screening programmes.

A key factor identified in our review was pre‐existing or comorbid psychological burden, which appears to significantly predict other psychological outcomes during LCS. Measuring participants' anxiety or distress at baseline participation in a LCS programme could therefore be critical in identifying participants who may experience psychological harm. For those with a cancer diagnosis, distress screening is part of the standard of care and there is a myriad of practice guidelines,^56^ though none extend to cancer screening. While measuring distress at every LCS visit may not be feasible or useful with the number of participants in a screening programme, strategies to enable participants to easily communicate distress, and for providers to easily identify it, at every touchpoint could be considered. As providers are often time‐poor during shared decision‐making discussions,^57^ approaches to assess participant distress without adding to clinician time burden may be useful. Pre‐completion of patient‐reported outcome or experience measures, or assessment of psychological outcomes by a nurse prior could be beneficial. Programme coordinators or ‘navigators’ have also been successful in providing engagement and support for participants during LCS, so involving them in distress screening and management could also be an option.^58^, ^59^ Appropriate triage and referral pathways for those experiencing psychosocial harm are also needed, which would require programme‐level set‐up and resourcing.^56^

Findings on factors and outcomes associated with risk perception in this review were varied. This likely reflects the complexity of risk comprehension and the integral role of personal, cultural and social biases. With this in mind, it is important to delineate between absolute (how likely are you to get lung cancer?) and comparative (how likely are you to get lung cancer *compared to someone similar to you*?) risk perception.^60^ Comparative risk perception has a direct social focus and therefore may be more tied up in cognitive biases and have other consequences for psychosocial outcomes.^61^ This difference was often not articulated in studies included in this review, with ‘risk perception’ used as an umbrella term for both concepts. Further research should investigate the differences in absolute and comparative risk perception on psychosocial outcomes specifically for LCS, especially considering the unique personal and social complexities around smoking behaviour and stigma in LCS. Findings from the Manchester Lung Health Check pilot suggest that provision of comparative (rather than absolute) risk could support better risk understanding, however it was flagged that further research was required to determine the optimal approach to risk communication in the LCS setting.^22^ The general population almost always significantly overestimate their cancer risk, and while evidence shows that risk information can help align perceptions closer to actual estimates, overestimations remain.^31^, ^61^ In addition, there is evidence that people who have stopped smoking underestimate their lung cancer risk due to perceived protective health benefits of having quit.^62^ These enduring inaccurate conceptualisations of risk are possible drivers behind the nil or positive impacts of personalised cancer risk information (PCRI) seen for psychological outcomes in the broader literature.^61^ This further necessitates research on how to effectively communicate risk in LCS, and to understand the true psychosocial effects.

The harms of stigma in cancer and non‐communicable respiratory diseases are well‐documented and include psychosocial burden, delays in medical help‐seeking behaviours and reduced participation in early detection activities.^63^, ^64^, ^65^ Lung cancer stigma is especially pervasive owing to decades of public focus on its links with smoking,^63^, ^66^ and the narrative of smoking being an individual ‘choice’ rather than an addiction. Most work to date on stigma in lung cancer has focused on those with a diagnosis, with less evidence on the impact of stigma in earlier stages of the lung cancer continuum or as early as the LCS pathway. Recent qualitative studies suggest that stigma is present during LCS and that it can act as a barrier to participation in screening,^37^, ^67^, ^68^ but there is limited evidence on the psychosocial consequences of stigma on screening, as identified by this review. Some studies referenced internalised shame, blame and guilt around smoking behaviour,^40^, ^42^ but there was little exploration of the impacts of interpersonal or societal level stigma.^63^ A detailed understanding of how and where stigma emerges during the LCS pathway is needed to design appropriate strategies to address it.

### 4.1 Limitations

There are limitations to address in this review. Critically, the findings from this review are based on a heterogeneous group of studies with varying methodological quality, with risk of bias assessed as moderate or high in 33 of 35 included studies. There was variation in study design and measurement of factors and outcomes of interest. Most studies were completed in trial settings or at a single site, limiting generalisability of conclusions to real‐world groups who would be participating in LCS. Selection bias and coverage bias were also common across quantitative studies.

## 5 CONCLUSION

This review provides the first comprehensive synthesis of participant‐level factors associated with psychological burden for LCS participants. These findings, despite the acknowledged limitations of the existing evidence, point to a clear association between certain individual factors and psychosocial impacts during LCS. Sociodemographic and health risk factors—for example, age, gender and smoking status—were the most examined in this review and are easily identifiable among participants in an LCS programme context. However, the evidence for these factors was mixed in terms of significant and non‐significant relationships reported. Some factors—particularly beliefs and expectations—were examined less frequently, and these factors may require more time and effort to elucidate during LCS, but could serve as more refined predictors of psychological harm and warrant further research. Importantly, the interplay of different participant factors in the LCS context should be considered when designing strategies to manage psychosocial experiences.

## AUTHOR CONTRIBUTIONS

**Kathleen McFadden:** Conceptualization (equal); data curation (lead); formal analysis (lead); investigation (equal); methodology (equal); project administration (lead); writing – original draft (lead). **Brooke Nickel:** Conceptualization (equal); data curation (equal); methodology (equal); supervision (equal); writing – review and editing (equal). **Nicole M. Rankin:** Conceptualization (equal); methodology (equal); supervision (equal); writing – review and editing (equal). **Tong Li:** Data curation (equal). **Chloe J. Jennett:** Data curation (equal). **Ashleigh Sharman:** Data curation (equal). **Samantha L. Quaife:** Conceptualization (equal); writing – review and editing (equal). **Nehmat Houssami:** Conceptualization (equal); methodology (equal); supervision (lead); writing – review and editing (equal). **Rachael H. Dodd:** Conceptualization (equal); methodology (equal); supervision (lead); writing – review and editing (equal).

## FUNDING INFORMATION

KM is supported by The Daffodil Centre Postgraduate Research Scholarship. NH is supported by the National Breast Cancer Foundation (NBCF) Chair in Breast Cancer Prevention grant (EC‐21‐001) and a NHMRC Investigator (Leader) grant (1194410). BN is supported by a NHMRC Investigator Emerging Leader Research Fellowship (1194108). TL is supported by a Cancer Institute NSW Early Career Fellowship (#2022/ECF1420). CJJ is supported by The Daffodil Centre Postgraduate Research Scholarship. SLQ is supported by a Barts Charity programme grant (G‐001522, MGU0461).

## CONFLICT OF INTEREST STATEMENT

The authors have no conflicts of interest to declare.

## Supporting information


Data S1.

## Data Availability

The data that supports the findings of this study are available in the supplementary material of this article.
