# Supplementary material for: Participant factors associated with psychosocial impacts of lung cancer screening: A systematic review
Source: Cancer Med. 2024 Aug 3;13(15):e70054. doi: 10.1002/cam4.70054 (PMC11297455; doi:10.1002/cam4.70054)
Supplement: Supplementary file 1 — Data S1. [file CAM4-13-e70054-s001.docx]

**Table S1. Ovid MEDLINE search strategy (last conducted 12 July 2023)**

| 1 | (psychosocial or psychological or social).mp. or Mental Health/ or Mental Disorders/ or *Patient reported outcome measures/ or (Patient Reported Outcome* or PROM*).mp. or exp Outcome Assessment, Health Care/ or exp Patient Outcome Assessment/ or exp Quality of Life/ or exp Health Surveys/ |
| --- | --- |
| 2 | (anxiet* or anxious*).mp. or Anxiety/ or exp Anxiety Disorders/ [mp=title, abstract, original title, name of substance word, subject heading word, floating sub-heading word, keyword heading word, organism supplementary concept word, protocol supplementary concept word, rare disease supplementary concept word, unique identifier, synonyms] |
| 3 | (depress* or dysphori* or dysthymi* or melanchol* or seasonal affective disorder*).mp. or Depression/ or exp Depressive Disorder/ |
| 4 | Stress, Psychological/ or (stress* or panic* or psychotrauma* or stress disorder*).mp. |
| 5 | Fear/ or (cancer adj3 fear).mp. or (cancer adj2 thoughts).mp. |
| 6 | Psychological Distress/ or distress.mp. |
| 7 | worry.mp. |
| 8 | (regret* or (decision* adj5 conflict*)).mp. |
| 9 | (coping or resilien*).mp. |
| 10 | (health?related quality of life or HRQOL or (quality adj2 life) or satisfaction).mp. |
| 11 | reassur*.mp. |
| 12 | ((self-efficacy or mastery or control) adj3 (feel* or felt or ability*)).mp. |
| 13 | 1 or 2 or 3 or 4 or 5 or 6 or 7 or 8 or 9 or 10 or 11 or 12 |
| 14 | (((positive or negative or indeterminate) adj2 results) or (abnormal or pulmonary nodule* or surveillance)).mp. |
| 15 | Risk Factors/ or risk factor*.mp. or exp Socioeconomic Factors/ or Sociodemographic Factors/ or exp Social Conditions/ or Income/ or Insurance/ or Healthcare Disparities/ or Sex Characteristics/ or (female or male or gender).mp. or Age Groups/ or (age or age group or old* or young*).mp. or Race Factors/ or (race or ethnicity or skin colo?r or migration background).mp. or Cross-cultural comparison/ or Educational Status/ or (education level or schooling).mp. or (living area or residence area or residence).mp. |
| 16 | Smoking/ or Smokers/ or Ex-Smokers/ or Tobacco/ or Tobacco Products/ or (smok* or tobacco or tobacco products or cigarette or pipe or cigar or nicotine or cotinine).mp. |
| 17 | ((cancer adj3 (experience* or perception or perceiv*)) or ((family or personal) adj2 history)).mp. |
| 18 | Health Belief Model/ or (health belief model or HBM or health behaviour or perception or susceptib* or invincibil* or denial or invulnerab* or vulnerab* or optimis*).mp. |
| 19 | (risk* or perceiv* or concern* or ambigu* or uncertain* or danger or chance or misfortun* or trust or distrust or mistrust or hope or guess or speculat* or secur* or insecur* or power or control or confidenc*).mp. |
| 20 | (stigma or prejudic* or nihilis*).mp. or exp Shame/ |
| 21 | trait.mp. |
| 22 | (delay or wait* or await*).mp. |
| 23 | (schedul* or appointment* or (service* adj2 accessib*)).mp. |
| 24 | Transportation of Patients/ or Health Services Accessibility/ or (proximit* or travel* or distance or transport* or access*).mp. or (journey* adj5 (car or bus or transit or transport* or public transport or train)).mp. or (travel* adj5 (distance or time)).mp. |
| 25 | Counseling/ or (counsel?ing or de?brief).mp. |
| 26 | Health Communication/ or (communication or disclos* or conversat* or discourse or verbal behavio?r or non-verbal behavio?r or interaction or interpersonal behavio?r or body language or sharing).mp. or report* style.mp. |
| 27 | Decision Making, Shared/ or (shared decision* or decision?aid or decision tool or decision?making).mp. or (decision adj3 support).mp. or (decision adj5 intervention).mp. |
| 28 | Patient Education as Topic/ or exp Consumer Health Information/ or education*.mp. |
| 29 | Psychosocial Intervention/ or Internet-Based Intervention/ or (behavio?ral intervention* or approach or strategy or intervention*).mp. |
| 30 | expect*.mp. |
| 31 | 14 or 15 or 16 or 17 or 18 or 19 or 20 or 21 or 22 or 23 or 24 or 25 or 26 or 27 or 28 or 29 or 30 |
| 32 | (low?dose computed tomography or (low?dose adj2 CT) or LDCT or lung cancer screening or ((computed tomography or chest CT or CT) adj2 lung)).mp. |
| 33 | 13 and 31 and 32 |

**Table S2. Summary of factors and outcomes examined by each study (n=35).**

| **Studies** | **Factors** | | | | **Outcomes** | | | | |
| --- | --- | --- | --- | --- | --- | --- | --- | --- | --- |
|  | **Socio-demographic** | **Health-related** | **Health beliefs** | **Other^a^** | **Psychological** | **Health beliefs** | **Decision-related** | **Smoking-related** | **Social** |
| Balata et al., 2020 |  | X |  |  |  |  |  | X |  |
| Barta et al., 2021 | X |  |  |  | X | X |  |  |  |
| Bold et al., 2022 | X | X |  |  |  |  |  | X |  |
| Brain et al., 2016 | X | X |  |  | X |  |  |  |  |
| Bunge et al., 2008 | X | X | X |  | X | X |  |  |  |
| Buttery et al., 2022 |  | X | X | X |  |  |  | X |  |
| Byrne et al., 2008 | X | X |  |  | X | X |  |  |  |
| Byrne et al., 2019 | X | X |  |  | X | X | X |  |  |
| Cordon et al., 2021 |  |  |  | X |  |  |  | X |  |
| Dunn et al., 2017 | X | X | X |  | X |  |  |  |  |
| Eberth et al., 2022 | X |  |  | X |  |  | X |  |  |
| Golden et al., 2020 |  |  | X |  | X |  |  | X |  |
| Golden et al., 2022 |  | X |  |  |  |  |  | X |  |
| Greene et al., 2019 |  | X |  |  | X | X |  |  |  |
| Hall et al., 2018 | X | X | X |  | X | X |  |  |  |
| Han et al., 2019 | X | X |  |  |  | X |  | X |  |
| Kaerlev et al., 2012 | X | X |  |  | X |  |  |  |  |
| Kathuria et al., 2020 |  |  | X |  |  |  |  | X |  |
| Kummer et al., 2020a |  | X | X | X | X | X |  | X | X |
| Kummer et al., 2020b | X | X |  |  | X |  |  |  |  |
| Lebrett et al., 2022 | X | X | X |  | X | X |  |  |  |
| Lee et al., 2021 | X | X |  |  | X |  |  | X |  |
| Lillie et al., 2017 | X | X |  |  | X | X |  |  |  |
| Nishi et al., 2021 | X | X |  |  |  |  | X |  |  |
| Olson et al., 2022 | X |  |  | X | X | X |  |  |  |
| Ostroff et al., 2001 |  |  | X |  |  |  |  | X |  |
| Quaife et al., 2021 | X | X |  |  | X | X |  | X |  |
| Taghizadeh et al., 2019 | X | X |  |  | X |  |  |  |  |
| Turner et al., 2021 | X | X | X |  | X | X |  | X |  |
| Van den Bergh et al., 2008 | X | X |  |  | X |  |  |  |  |
| Van den Bergh et al., 2010a | X | X |  |  | X |  |  |  |  |
| Van den Bergh et al., 2010b |  |  |  | X | X | X |  |  |  |
| Van den Bergh et al., 2011 | X | X |  |  | X |  |  |  |  |
| Williams et al., 2022 | X | X | X |  |  |  |  | X |  |
| Zeliadt et al., 2015 |  |  | X |  | X |  |  |  |  |
| **Totals** | **24** | **26** | **12** | **6** | **24** | **14** | **3** | **14** | **1** |

a. Including: Social factors; Informed decision-making and knowledge; Responses to COVID-19

**Table S3. Study quality assessed using JBI critical appraisal checklist for quasi-experimental studies^.**

| **Study (author, year)** | **1. Is it clear in the study what is the ‘cause’ and what is the ‘effect’ (i.e. there is no confusion about which variable comes first)?** | **2. Were the participants included in any comparisons similar?** | **3. Were the participants included in any comparisons receiving similar treatment/care, other than the exposure or intervention of interest?** | **4. Was there a control group?** | **5. Were there multiple measurements of the outcome both pre and post the intervention/ exposure?** | **6. Was follow up complete and if not, were differences between groups in terms of their follow up adequately described and analyzed?** | **7. Were the outcomes of participants included in any comparisons measured in the same way?** | **8. Were outcomes measured in a reliable way?** | **9. Was appropriate statistical analysis used?** | **Overall RoB*** |
| --- | --- | --- | --- | --- | --- | --- | --- | --- | --- | --- |
| Brain et al., 2016 | Yes | Yes | Yes | Yes | Unclear | Unclear | Yes | Yes | No | **Moderate** |
| Bunge et al., 2008 | Yes | Yes | Yes | No | No | No | Yes | Unclear | Yes | **High** |
| Byrne et al., 2008 | Yes | Yes | Yes | No | Unclear | Yes | Yes | Unclear | Yes | **Moderate** |
| Dunn et al., 2017 | Yes | Yes | Yes | No | No | Unclear | Yes | No | Yes | **High** |
| Kummer et al., 2020b | Yes | Yes | Yes | Yes | Unclear | Unclear | Yes | Yes | Yes | **Moderate** |
| Lee et al., 2021 | Unclear | Yes | Yes | No | No | No | Yes | No | No | **High** |
| Taghizadeh et al., 2019 | Yes | Yes | Yes | No | No | No | Yes | Yes | Yes | **High** |
| Van den Bergh et al., 2008 | Yes | Yes | Yes | No | Yes | Yes | Yes | Unclear | Yes | **Moderate** |
| Van den Bergh et al., 2010a | Yes | Yes | Yes | No | Unclear | No | Yes | Yes | Yes | **High** |
| Van den Bergh et al., 2010b | Yes | Yes | Yes | No | Unclear | No | Yes | Unclear | Yes | **High** |
| Van den Bergh et al., 2011 | Yes | Yes | Yes | Yes | Unclear | No | Yes | Yes | Yes | **Moderate** |
| Williams et al., 2022 | Yes | Yes | Yes | No | No | Yes | Yes | Yes | Yes | **Moderate** |

*The criteria for overall risk of bias assessment were: Low: the study meets all criteria or has 1 “Unclear” item; Moderate: the study has 2-3 "Unclear" OR 1 “No” and 0-2 “Unclear” OR 2 “No”; OR High: the study has 1 "No" and 3+ "Unclear" OR 2 “No” and 1+ “Unclear” OR 3+ “No” OR 4+ "Unclear". “Not applicable” wasn’t used to determine risk of bias category. All studies, regardless of their quality, were included in the analysis.

^Tufanaru C, Munn Z, Aromataris E, Campbell J, Hopp L. Chapter 3: Systematic reviews of effectiveness. In: Aromataris E, Munn Z (Editors). JBI Manual for Evidence Synthesis. JBI, 2020. Available from https://synthesismanual.jbi.global

**Table S4. Study quality assessed using JBI critical appraisal checklist for prevalence studies^ (cross-sectional studies).**

| **Study (author, year)** | **1. Was the sample frame appropriate to address the target population?** | **2. Were study participants sampled in an appropriate way?** | **3. Was the sample size adequate?** | **4. Were the study subjects and the setting described in detail?** | **5. Was the data analysis conducted with sufficient coverage of the identified sample?** | **6. Were valid methods used for the identification of the condition?** | **7. Was the condition measured in a standard, reliable way for all participants?** | **8. Was there appropriate statistical analysis?** | **9. Was the response rate adequate, and if not, was the low response rate managed appropriately?** | **Overall RoB*** |
| --- | --- | --- | --- | --- | --- | --- | --- | --- | --- | --- |
| Balata et al., 2020 | Yes | Yes | Unclear | Yes | Unclear | No | Yes | Yes | Yes | **Moderate** |
| Barta et al., 2021 | Unclear | Yes | Unclear | Yes | Unclear | Unclear | Yes | Yes | No | **High** |
| Bold et al., 2022 | Unclear | Yes | Unclear | Unclear | No | No | Yes | Yes | Unclear | **High** |
| Byrne et al., 2019 | No | Yes | No | Yes | No | Unclear | Yes | Yes | No | **High** |
| Cordon et al., 2021 | Unclear | Yes | Unclear | No | Unclear | No | Yes | Yes | Unclear | **High** |
| Eberth et al., 2022 | Unclear | Yes | Unclear | Yes | Unclear | Yes | Yes | Yes | No | **High** |
| Hall et al., 2018 | Unclear | Yes | Unclear | Yes | Unclear | Unclear | Yes | Yes | No | **High** |
| Han et al., 2019 | No | Yes | Unclear | Unclear | Unclear | No | Yes | Yes | Yes | **High** |
| Kaerlev et al., 2012 | No | Yes | Yes | Unclear | Yes | Yes | Unclear | Yes | Yes | **Moderate** |
| Lebrett et al., 2022 | No | Yes | Unclear | Yes | Unclear | Yes | Yes | Yes | Yes | **Moderate** |
| Lillie et al., 2017 | No | Yes | Unclear | Yes | Unclear | No | Yes | Yes | Unclear | **High** |
| Nishi et al., 2021 | Unclear | Yes | Unclear | Yes | Unclear | Yes | Unclear | Yes | No | **High** |
| Ostroff et al., 2001 | Unclear | Yes | Unclear | Yes | Yes | Unclear | Yes | Yes | Yes | **Moderate** |
| Quaife et al., 2021 | Yes | Yes | Yes | Yes | Yes | Yes | Yes | Yes | Yes | **Low** |
| Turner et al., 2021 | Yes | Yes | Yes | Yes | Unclear | No | Yes | Yes | Unclear | **Moderate** |

*The criteria for overall risk of bias assessment were: Low: the study meets all criteria or has 1 “Unclear” item; Moderate: the study has 2-3 "Unclear" OR 1 “No” and 0-2 “Unclear” OR 2 “No”; OR High: the study has 1 "No" and 3+ "Unclear" OR 2 “No” and 1+ “Unclear” OR 3+ “No” OR 4+ "Unclear". “Not applicable” wasn’t used to determine risk of bias category. All studies, regardless of their quality, were included in the analysis.

^Munn Z, Moola S, Lisy K, Riitano D, Tufanaru C. Methodological guidance for systematic reviews of observational epidemiological studies reporting prevalence and incidence data. Int J Evid Based Healthc. 2015;13(3):147–153.

**Table S5. Study quality assessed using JBI critical appraisal checklist for qualitative studies^.**

| **Study (author, year)** | **1. Is there congruity between the stated philosophical perspective and the research methodology?** | **2. Is there congruity between the research methodology and the research question or objectives?** | **3. Is there congruity between the research methodology and the methods used to collect data?** | **4. Is there congruity between the research methodology and the representation and analysis of data?** | **5. Is there congruity between the research methodology and the interpretation of results?** | **6. Is there a statement locating the researcher culturally or theoretically?** | **7. Is the influence of the researcher on the research, and vice- versa, addressed?** | **8. Are participants, and their voices, adequately represented?** | **9. Is the research ethical according to current criteria or, for recent studies, and is there evidence of ethical approval by an appropriate body?** | **10. Do the conclusions drawn in the research report flow from the analysis, or interpretation, of the data?** | **Overall RoB*** |
| --- | --- | --- | --- | --- | --- | --- | --- | --- | --- | --- | --- |
| Buttery et al., 2022 | Yes | Yes | Yes | Yes | Yes | No | No | Yes | Yes | Yes | **Moderate** |
| Cordon et al., 2021 | Yes | Yes | Yes | Yes | Yes | No | Unclear | Yes | Yes | Yes | **Moderate** |
| Golden et al., 2020b | Yes | Yes | No | Yes | Yes | Yes | Yes | Yes | Yes | Yes | **Moderate** |
| Golden et al., 2022 | Yes | Yes | No | Yes | No | Yes | Yes | Yes | Yes | Yes | **Moderate** |
| Greene et al., 2019 | Yes | Yes | Unclear | Yes | Yes | No | Unclear | Yes | Yes | Yes | **Moderate** |
| Kathuria et al., 2020 | Yes | Yes | Yes | Yes | Yes | No | Yes | Yes | Yes | Yes | **Moderate** |
| Kummer et al., 2020a | Yes | Yes | Yes | Yes | Yes | No | Unclear | Yes | Yes | Yes | **Moderate** |
| Olson et al., 2022 | Yes | Yes | Yes | Yes | Yes | Yes | Yes | Yes | Yes | Yes | **Low** |
| Zeliadt et al., 2015 | Yes | Yes | Unclear | Yes | Yes | No | No | Yes | Yes | Yes | **High** |

*The criteria for overall risk of bias assessment were: Low: the study meets all criteria or has 1 “Unclear” item; Moderate: the study has 2-3 "Unclear" OR 1 “No” and 0-2 “Unclear” OR 2 “No”; OR High: the study has 1 "No" and 3+ "Unclear" OR 2 “No” and 1+ “Unclear” OR 3+ “No” OR 4+ "Unclear". “Not applicable” wasn’t used to determine risk of bias category. All studies, regardless of their quality, were included in the analysis.

^Lockwood C, Munn Z, Porritt K. Qualitative research synthesis: methodological guidance for systematic reviewers utilizing meta-aggregation. Int J Evid Based Healthc. 2015;13(3):179–187.
